# The relation between receptive grammar and procedural, declarative, and working memory in specific language impairment

**DOI:** 10.3389/fpsyg.2015.01090

**Published:** 2015-08-03

**Authors:** Gina Conti-Ramsden, Michael T. Ullman, Jarrad A. G. Lum

**Affiliations:** ^1^School of Psychological Sciences, The University of Manchester, Manchester, UK; ^2^Department of Neuroscience, Georgetown University, Washington, DC, USA; ^3^School of Psychology, Deakin University, Melbourne, VIC, Australia

**Keywords:** memory, compensation, grammar, receptive grammar, specific language impairment, working memory, declarative memory, procedural memory

## Abstract

What memory systems underlie grammar in children, and do these differ between typically developing (TD) children and children with specific language impairment (SLI)? Whilst there is substantial evidence linking certain memory deficits to the language problems in children with SLI, few studies have investigated multiple memory systems simultaneously, examining not only possible memory deficits but also memory abilities that may play a compensatory role. This study examined the extent to which procedural, declarative, and working memory abilities predict receptive grammar in 45 primary school aged children with SLI (30 males, 15 females) and 46 TD children (30 males, 16 females), both on average 9;10 years of age. Regression analyses probed measures of all three memory systems simultaneously as potential predictors of receptive grammar. The model was significant, explaining 51.6% of the variance. There was a significant main effect of learning in procedural memory and a significant group × procedural learning interaction. Further investigation of the interaction revealed that procedural learning predicted grammar in TD but not in children with SLI. Indeed, procedural learning was the only predictor of grammar in TD. In contrast, only learning in declarative memory significantly predicted grammar in SLI. Thus, different memory systems are associated with receptive grammar abilities in children with SLI and their TD peers. This study is, to our knowledge, the first to demonstrate a significant group by memory system interaction in predicting grammar in children with SLI and their TD peers. In line with Ullman’s Declarative/Procedural model of language and procedural deficit hypothesis of SLI, variability in understanding sentences of varying grammatical complexity appears to be associated with variability in procedural memory abilities in TD children, but with declarative memory, as an apparent compensatory mechanism, in children with SLI.

## Introduction

Specific language impairment (SLI) is a neurodevelopmental disorder that affects around 3–7% of children (Tomblin et al., 1997; American Psychiatric Association, 2000), and can have serious impacts on a child’s life (Conti-Ramsden et al., 2012). Its diagnosis requires a child to have difficulties understanding and/or producing language despite having adequate hearing, and intelligence scores within the normal range. Moreover, children with SLI have a hallmark phenotype: poor grammatical abilities.

Although the term SLI has been used extensively in the research literature, the label is increasingly disfavoured. The “Specific” in SLI implies that the difficulties these children experience are limited to language, with everything else being otherwise “normal.” “Specific” also implies a clear discrepancy between verbal abilities and non-verbal IQ, such that children with SLI should not exhibit low non-verbal IQ. Both these assumptions have been heavily criticized (Ullman and Pierpont, 2005; Montgomery et al., 2010; Bishop, 2014; Reilly et al., 2014b). It also needs to be noted that the definition of SLI continues to rely more on exclusionary criteria (e.g., the lack of hearing and intelligence deficits, or the absence of co-morbidity with other disorders such as autism) than on a specification of the difficulties. However, this state of affairs is no longer tenable, and a swell of discussion and consultation have, ensued in academic venues (see Bishop, 2014; Conti-Ramsden, 2014; Reilly et al., 2014a) as well as in social media (see, for example, https://twitter.com/deevybee). Nevertheless, it will take time before a new term or terms are widely accepted. In the absence of such a new agreed-upon diagnostic label, we continue to use the term SLI, while fully acknowledging its limitations.

We now turn to what is known about the relationship between memory and language in SLI. To date, most theoretical and empirical work examining this relationship has focused on short-term and working memory (e.g., Ellis Weismer et al., 1999; Marton and Schwartz, 2003; Archibald and Gathercole, 2006a, 2007). This literature suggests that children with SLI generally perform significantly worse than typically developing (TD) children on tests of verbal short-term or working memory, i.e., those that require the temporary storage of verbal information (short-term memory) and the simultaneous processing of this information (working memory), such as non-word repetition or backward digit recall. In contrast, evidence suggests that non-verbal short-term and non-verbal working memory remain largely intact in SLI (Archibald and Gathercole, 2006b; Alloway and Archibald, 2008).

Impairments in verbal working memory have been proposed to underlie grammatical difficulties in SLI (Adams and Gathercole, 1996; Ellis Weismer et al., 1999). Baddeley and Hitch’s model of working memory (Baddeley and Hitch, 1974; Baddeley, 2003) suggests that during sentence processing the “phonological loop” holds incoming auditory information until processing can be completed (Cowan’s model has close corollaries; Cowan, 2012). This seems to occur during the processing of complex grammatical structures (Montgomery and Evans, 2009; Montgomery et al., 2009). Montgomery (1995), in his study of 8-year old children, found significant correlations between measures of verbal working memory and tasks involving receptive grammar for both children with SLI and their TD peers. These findings have been replicated by Norbury et al. (2002) using standardized tests of comprehension of grammar, i.e., language comprehension requiring grammatical knowledge. Nonetheless, it should be noted that investigations examining verbal working memory-comprehension relationships have mainly focused on adults (Gathercole and Baddeley, 2014); with children, findings have been more mixed, particularly with TD children. Thus, the role of working memory in sentence comprehension in children remains unclear (see Kidd, 2013, for a review). Moreover, correlations between measures of expressive grammar and those of working memory have been less consistent. For example, a number of investigators have found that verbal working memory tasks correlate weakly with expressive tasks involving grammar such as past-tense elicitation (Botting and Conti-Ramsden, 2001; Norbury et al., 2001; Bishop et al., 2006).

Our own group has found that working memory abilities do not correlate significantly with grammar in either SLI children or their TD peers (Lum et al., 2012). However, the measure of grammar used in these analyses was a composite of expressive and receptive tasks. This may have hidden any association of working memory on grammar that might only be evident for receptive grammar in children with SLI. Overall, given the small as well as mixed literature, it would be of interest to further examine the relationship between verbal working memory and grammar in children with SLI and their TD peers, focusing particularly on receptive grammar given that it has shown the most consistent correlations.

However, not all research on memory systems in SLI has focused on working memory. The procedural deficit hypothesis (PDH) posits that SLI may be largely explained by abnormalities of brain structures underlying procedural memory, in particular portions of frontal/basal-ganglia circuits (Ullman, 2004; Ullman and Pierpont, 2005). According to the PDH, this may also explain the impairment of other functions that rely on portions of these brain structures, such as aspects of working memory.

The PDH is an extension of a wider neurobiologically-motivated theoretical model of normal and disordered language acquisition developed by Michael Ullman and colleagues (Ullman, 2001a, 2004, in press), the declarative/procedural (DP) model. This model posits that procedural memory generally underlies the learning, storage and use of important aspects of grammar, in particular (at least) implicit rule-governed grammatical knowledge that involves (hierarchical) sequencing. Hence, deficits in procedural memory should lead to grammatical impairments, as posited by the PDH for SLI (Ullman and Pierpont, 2005). Declarative memory is posited to be critical for lexical memory. However, the DP model hypothesizes that declarative memory can also perform many grammatical functions, and thus often plays a compensatory role when procedural memory is not fully functioning. This is possible because declarative memory is highly flexible, and thus can learn, store and process knowledge to accomplish grammatical functions normally carried out by procedural memory. Moreover, it can do so in a variety of ways, such as chunking (e.g., storing “the cat” or “walked” rather than composing them from their parts) or learning explicit (or even implicit) rules (Ullman, 2004, in press). The PDH specifically posits such types of declarative-memory based compensation for grammar in SLI (Ullman and Pierpont, 2005; Ullman and Pullman, 2015).

Since the PDH was original proposed (Ullman, 2004; Ullman and Pierpont, 2005), an increasing amount of empirical research has examined procedural memory in children with SLI. Importantly, a recent meta-analysis suggests, consistent with the predictions of the PDH, that SLI is indeed associated with significantly poorer procedural learning than TD individuals, as tested by the serial reaction time (SRT) task (Lum et al., 2014). Note that almost all studies of procedural memory in SLI have used non-verbal stimuli (e.g., the SRT task). However, learning tasks that involve verbal stimuli that seem to depend on procedural memory, such as the word segmentation task, also have shown deficits in SLI (Evans et al., 2009; Karuza et al., 2013).

In contrast, declarative memory has been much less well studied in SLI. Nevertheless, on the whole evidence seems to suggest that learning in this system is spared for non-verbal information, and even for verbal information once factors such as working memory abilities are held constant (Lum et al., 2012, 2014). Indeed, it appears that only children with SLI with poor verbal working memory show impairments on verbal declarative memory tasks (Lum et al., 2015). Importantly, there has also been relatively little investigation on the association between grammar and declarative memory in SLI, and on the possible compensatory role of this memory system for the grammatical deficits found in the disorder. Although some behavioral evidence (e.g., from frequency effects) and electrophysiological evidence (from Event-Related Potentials) supports such compensation (for reviews, see Ullman and Pierpont, 2005; Ullman and Pullman, 2015), we are only aware of one study examining associations between grammar and declarative memory, carried out by our group (Lum et al., 2012).

In that study we examined working, declarative and procedural memory in children with SLI and their TD peers (Lum et al., 2012). The children with SLI were impaired at verbal (but not non-verbal) short-term memory and working memory, and at (non-verbal) procedural learning (as in most studies, verbal procedural memory was not examined), but were spared at learning non-verbal information in declarative memory, as well as verbal information once language or working memory deficits were controlled for. We also found an intriguing pattern of correlations between grammatical abilities (as measured by a composite of expressive and receptive grammar; see above) and measures of these memory systems. Grammatical abilities correlated significantly with procedural learning in TD children but not in children with SLI. In contrast, in children with SLI (but not in TD children) grammar correlated with declarative memory but not with procedural memory.

These correlational findings warrant further investigation. Although they are clearly informative, it is important to emphasize that such correlations do not allow for the simultaneous examination of the predictiveness of all three memory systems on grammar, that is, holding the influence of the others constant. The present study addresses this gap.

Specifically, it moves the field forward in three specific ways. First, we used multiple regression analyses to simultaneously examine the influence of procedural, declarative and working memory on grammar in SLI and TD. Crucially, this modeling approach allows us to directly test whether and how each of the three memory systems might be associated with grammatical abilities in children with SLI and their TD peers, while holding the influence of the other memory systems constant and additionally examining whether and how their influence on grammar might differ between the two groups. Holding constant the influence of each memory system is important because the memory systems may interact. For example, working memory seems to be closely linked to declarative memory; as mentioned above, impairments of verbal declarative memory in SLI appear to only occur in the presence of verbal working memory deficits (Lum et al., 2015). Second, we focus on receptive grammar, given that previous research suggests stronger links between verbal working memory and receptive rather than expressive measures of grammar in SLI. Third, whereas most previous research has examined how deficits of memory (in particular deficits of working and procedural memory) might contribute to the grammatical impairments in SLI, we examine the potential *positive* contributions of declarative memory to language in children with SLI.

Thus, in this study we used multiple regression analyses to examine the roles of procedural, declarative and working memory on receptive grammar in children with SLI and their TD peers. In line with the DP model and the PDH, we expected procedural memory (but not declarative memory) to significantly predict grammar in TD children, and for declarative memory (but not procedural memory) to predict grammar in children with SLI. Given that the DP model and the PDH specify that other functions that rely on frontal/basal-ganglia circuitry may also be impaired in SLI, working memory was also expected as a possible predictor of grammar, at least for children with SLI. This study is, to our knowledge, the first to examine whether memory systems not only predict grammar in SLI and TD, but also whether they interact with group—that is, whether the measures of the memory systems show differential predictiveness for children with SLI and TD children.

## Materials and Methods

### Participants

The children participating in this study were a subgroup of children that participated in Lum et al. (2012). Given the focus on receptive grammar, we excluded 6 children with SLI who had receptive language skills above a standard score of 85 in the CELF-4 UK (1 SD from the mean). We also excluded 5 TD children who had receptive language skills –1 SD or below. This resulted in 45 children with SLI (mean age 9;10 years; 30 males, 15 females) and 46 TD children (mean age 9;10 years; 30 males, 16 females). Discrepancy between verbal scores and performance IQ scores was not a requirement for ascertaining the SLI phenotype. As discussed in the introduction, there is no empirical evidence to support a mismatch criterion for SLI participant selection (e.g., at least 1 SD difference between verbal and non-verbal scores). All children were recruited from schools in consultation with classroom teachers. These professionals advised the research team on potential participants based on their opinion as to whether children had poor versus good language. Subsequently, language abilities were measured directly using the core language score (CLS) of the Clinical Evaluation of Language Fundamentals—4th Edition, UK Standardization (CELF-4 UK, Semel et al., 2003). All children with SLI and TD children had Performance IQ (PIQ) scores within the typical range, i.e., no less than 1 SD below the mean on the Wechsler Abbreviated Scale of Intelligence (WASI, Wechsler, 1999). Table 1 shows the age (in whole months), CLS and PIQ scores of children with SLI and TD children.

**TABLE 1:** **Age and standardized tests: summary scores and comparisons between the SLI and TD groups**.

	**SLI (*n* = 45)**			**TD (*n* = 46)**			**Comparison**		
**Variable**	***M***	**SD**	**Range**	***M***	**SD**	**Range**	***t***	***p***	**Cohen’s *d***
Age (months)	118	9.2	103–137	118	8.6	102–137	0.22	0.826	–
CLS	70.7	8.5	46–84	99.9	6.2	90–117	18.79	<0.001	3.93
PIQ	97.2	7.0	85–110	99.9	7.8	85–115	1.78	0.079	0.36

SLI, children with Specific Language Impairment; TD, typically developing children; M, Mean; SD: Standard deviation; CLS, Core Language Score of the Clinical Evaluation of Language Fundamentals; PIQ, Performance IQ.

### Materials and Methods

#### Predictor Variables: Measures of Different Memory Systems

The memory measures used in this study are a subset of those used and described in detail in Lum et al. (2012). They are outlined here.

***Procedural memory***

Unlike for working and declarative memory, no verbal or auditory procedural memory task was given to participants. This was, first of all, because auditory SRT Tasks usually require participants to discriminate between tones of different frequencies (e.g., Zhuang et al., 1998), which might be problematic for children with SLI (McArthur and Bishop, 2004; Hill et al., 2005). A similar argument can be made for the word segmentation task (Evans et al., 2009). Additionally, our focus on a visuo-spatial SRT Task was not considered to be problematic for examining the contribution of procedural memory to language functioning, since, as we have seen above, the classic (and much more widely studied) visuo-spatial version of this task has been shown to be impaired in SLI (unlike non-verbal working and declarative memory tasks), and has been found to correlate with grammar, in TD children; see above.

Procedural memory was assessed using a version of Nissen and Bullemer’s (1987) SRT task. This task is designed to test implicit visuo-spatial sequence learning in procedural memory. In SRT tasks, participants are typically asked to press one of four response buttons, each of which matches the location of a visual stimulus presented on a computer monitor. Unbeknownst to participants, the visual stimulus follows a predefined sequence. After multiple exposures to the sequence, a random pattern of visual stimuli (rather than the predefined sequence) is presented. In neurologically intact children and adults, reaction times (RTs), which are the principal dependent measure of interest in SRT tasks, typically decrease during the repeated presentation of the sequence, and increase from the final sequence presentations to the random pattern (e.g., Nissen and Bullemer’s (1987); Thomas et al., 2004). This RT increase is taken as evidence that knowledge of the sequence has been learned.

To control for subject variability in motor speed, each child’s RTs were converted to z-scores referenced to the median and SD across all correct trials for that child (Thomas et al., 2004; Lum et al., 2012). Normalizing data in this way effectively ensured that all children’s shortest RTs have approximately the same value, and similarly for their longest RTs. For example, if the longest RT for one child was 5000 ms and longest for another was 1000 ms, after z-normalizing the values for both children might be 5 (i.e., 5 SD above the median of their overall RTs). Finally, we also addressed potential attention lapses in this task. This was considered important since the task was relatively long, with five blocks each of 90 trials, totalling to about 13 min. To deal with this concern, we deleted data points for each child whose RTs were 3 SD or more above his/her mean RT (Lum et al., 2012). Subsequently, z-scores referenced to the mean and standard deviation of the entire sample were then calculated (see section on use of z-scores below for details).

***Declarative memory***

The Children’s Memory Scales (CMS, Cohen, 1997) Word Pairs and Stories subtests were used to examine the learning and retrieval of verbal information in declarative memory. Only verbal (and not visual) declarative memory was examined. Previous findings by our group (Lum et al., 2012) revealed that whereas verbal declarative memory correlated with lexical abilities both in children with SLI and TD children, as well as grammar in children with SLI (see Introduction), visual declarative memory did not correlate with either lexical or grammatical abilities in either group. Thus focusing on verbal declarative memory maximized the likelihood of obtaining the correlations of interest.

The Word Pairs subtest assesses how well children learn a list of semantically unrelated word pairs, then recall as well as recognize them at a later point in time. First, children are presented with a list of 14 semantically unrelated word pairs (e.g., rice-chair). Immediately after, children are asked to provide the second word from memory. This procedure is repeated for three trials (Learning), and then, the children are asked to recall both words of each pair from memory (Immediate Recall). After other subtests on the CMS have been administered (typically about 30 min), children are again asked to recall the full list of word pairs (Delayed Recall). This is followed by the presentation of the 14 word pairs along with 14 distracter pairs, with the children indicating whether or not they recognize the target pairs from earlier in the test (Delayed Recognition). The Stories subtest assesses how well children can recall and recognize a short narrative comprising five to six sentences following a single exposure.

For analyses, the raw scores from each subtest were converted to a *z*-score referenced to the mean and standard deviation of the entire sample, see below for details. A composite measure was then computed by summing *z*-transformed subtests.

***Working memory***

Working memory functioning was assessed with the Working Memory Test Battery for Children (WMTB-C, Pickering and Gathercole, 2001). For the purposes of the present study, subtests assessing verbal working memory and “phonological loop” were used.

The WMTB-C has three subtests designed to assess verbal working memory: Listening Recall, Counting Recall and Backward Digits Recall. Common to these subtests is that children are presented with verbal information and are then required to temporarily store the information and engage in some additional processing on it. In the Listening Recall subtest, children are auditorily presented with sentences. Their task is to provide a true/false judgment about the sentence’s semantics and then recall the sentence’s final word. The task increases in difficulty as children are presented with an increasing number of sentences. After each sentence presentation a true/false judgment is made. Then, following presentation of a block of sentences, all sentence-final words are recalled. On the Counting Recall subtest, children are asked to count out loud arrays of dots presented in cards in the stimulus booklet. Immediately after counting, children are asked to recall how many dots were there. Children count and recall increasing number of dot arrays (from one dot array card followed by recall, two dot array cards followed by recall, up to a maximum of seven dot array cards, followed by recall). On the Backward Digit Recall subtest a string of digits are presented. The task is to repeat the string in reverse order.

The phonological loop was designed to be assessed by four subtests: Digit Recall, Word List Recall, Non-word List Recall, and Word List Matching. On all of these subtests verbal information is presented and the task is to temporarily store the presented information. On the Digit Recall, Word List Recall, and Non-word List Recall, children are respectively presented with an increasing number of digits, real words or non-words. Following each presentation the children are asked to recall the set of items. In the Word List Matching task, a series of words, beginning with two words and adding one word at each successive level, is presented to the child. The same words, but sometimes in a different order, are then presented again, and the child is asked to determine if the second list is in the same or different order as the first list.

These subtests were selected because they assess verbal aspects of working memory. It was considered important to focus on verbal working memory because (a) it has been proposed that deficits of verbal working memory (and not visual-spatial working memory) underlie language difficulties in SLI (Adams and Gathercole, 1996); and (b) significant correlations have been found between verbal working memory and receptive grammar in children with SLI and in TD children (Montgomery, 1995). See the Introduction for more details.

For the analyses, raw scores were first transformed to z-scores referenced to the mean and standard deviation of the entire sample; see below for details. A composite verbal working memory measure was created by summing *z*-scores from each subtest.

#### Outcome Variable: Receptive Grammar

The Test for Reception of Grammar 2nd Edition (TROG-2, Bishop, 2003) was used. The TROG-2 consists of 80 sentences evenly divided into 20 blocks. Children are presented with a sentence and asked to point to the matching picture from four possible options. For example, when presented with the stimulus sentence “The man that is eating is looking at the cat”, the four pictorial options include the correct pictorial representation of the sentence plus three incorrect options: the man eating but not looking at the cat, the man looking at the cat and the cat is eating, and the cat looking at the man who is not eating. As children progress through each block, increasingly more complicated syntactic structures are presented. A child does not pass a block if s/he failed at least one item on the block. Testing is discontinued if the child fails five consecutive blocks. The total number of correct items was used as the dependent variable in the analyses. The distribution of scores was found to be negatively skewed; a reflected square root transformation was applied to the data. Furthermore, z-scores referenced to the mean and standard deviation of the entire sample were then calculated.

#### The Use of z-scores

The decision to use z-scores in our analyses had two motivations. First, the working memory and declarative memory measures were created by combining scores from multiple subtests. Summing z-transformed raw scores ensures that each subtest is equally weighted in the composite variable (e.g., Ackerman and Cianciolo, 2000). Without this transformation a subtest that has a larger standard deviation will be weighted more in the composite variable. Second, by presenting group performance using z-score units, one is better able to directly evaluate group differences for the memory and language variables as they are, in effect being compared using the same scale. The procedure for referencing the z-scores to the mean and standard deviation of the entire sample involved pooling data from all children in a particular group (e.g., the SLI group) to calculate the referenced z-scores for each child in that group.

**FIGURE 1:**
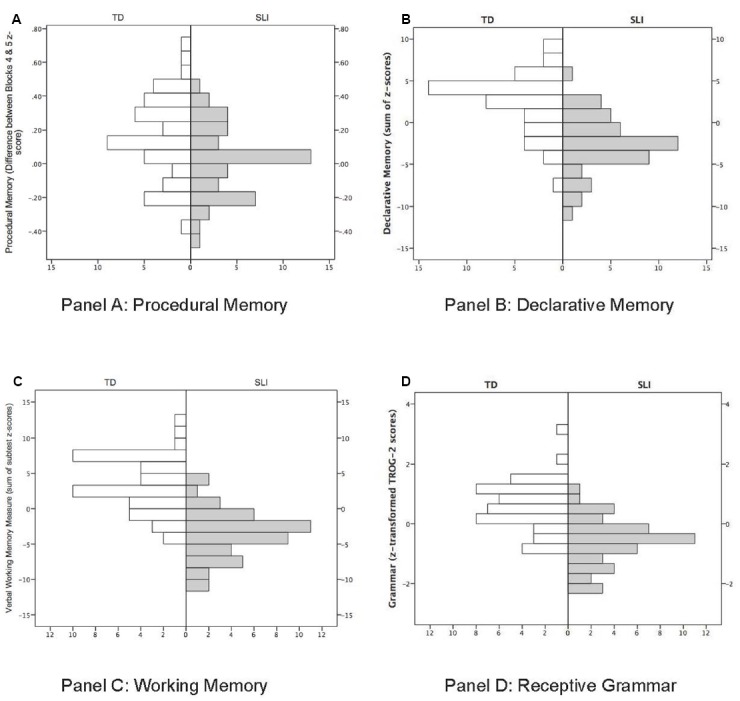
**Histograms showing distribution of scores for the measures of procedural memory (A), declarative memory (B), working memory (C), and receptive grammar (D) reported by group.** Shaded bars show the distributions for the SLI group (right side of each panel), while white bars show the distributions for the TD group (left side of each panel).

Note that the z-transformations of the different measures did not alter the distributions of scores. Prior to converting raw scores to z-scores the distribution of the variables was found to be normally distributed (except for TROG-2 scores which were skewed, but were normally distributed after application of a reflected square root transformation; see above). Furthermore, the correlation between the raw and z-score transformed scores was high (*r*’s > 0.9). Thus the z-transformation did not affect the ranking of participants’ scores (e.g., children with high grammatical scores prior to transformation still obtained high scores after transformation). Distributional information for the SLI and TD groups for each measure is summarized in the histograms presented in Figure 1.

### Procedure

The assessment battery was administered to participants over an average of five sessions in order not to interfere with their school schedule. Only one memory task was presented per session. The order of presentation of tasks was randomized across participants. Each test session was separated by a 7–14 day interval; all sessions were completed within a 3 month period. Ethical approval for the study was obtained from The University of Manchester, and informed written consent was obtained from the children’s parents or legal guardians.

## Results

Performance of the two groups of children on each of the variables of interest is shown in Table 2. Children with SLI had significantly lower scores on all predictor variables, that is, measures of procedural memory, declarative memory, and working memory. Children with SLI also had significantly lower scores on receptive grammar as measured by the TROG-2. It should be noted the average standard score for the SLI group was, as expected, more than 1 SD below the normative mean on the TROG-2 (*M* = 81.69, SD = 14.41). In contrast, the average standard score for the TD group was slightly above the normative mean (*M* = 102.57, SD = 11.34). Thus, as expected, the children with SLI were impaired on the measure of receptive grammar used in this study.

**TABLE 2:** **Memory and grammar measures: Summary scores (z-transformed) and comparisons between the SLI and TD groups**.

	**SLI**				**TD**				**Comparison**		
**Variable**	***M***	**SD**		**Range**		***M***	**SD**		**Range**		***t***	***p***	**Cohen’s *d***
Memory measures													
Procedural memory (SRT Task)	–0.30	0.86	–2.30	–	1.40	0.30	1.04	–1.97	–	2.45	3.00	0.003	0.63
Declarative memory (CMS)	–2.67	3.34	–11.62	–	5.05	2.61	3.53	–6.96	–	9.98	7.34	<0.001	1.54
Working memory (WMTB-C)	–3.36	3.57	–10.98	–	4.93	3.28	3.83	–4.28	–	11.85	8.54	<0.001	1.79
Language measure													
Receptive grammar (TROG-2)	–0.54	1.13	–3.67	–	0.84	0.53	0.41	–0.49	–	1.15	5.97	<0.001	1.25

SLI, children with Specific Language Impairment; TD, typically developing children; M, Mean; SD, Standard deviation; SRT, serial Reaction Time; CMS, Children’s Memory Scale; WMTB-C, Working Memory Test Battery for Children; TROG-2, Test for Reception of Grammar 2nd Edition.

**FIGURE 2:**
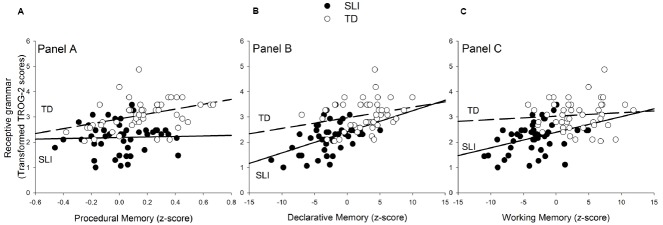
**Scatterplots showing the relationship between TROG-2 scores and the measures of procedural memory (A), declarative memory (B), and working memory (C) for SLI and TD groups.** Dashed line plots regression equation for TD group and unbroken for SLI group. Recall that the procedural memory task is a non-verbal task.

The next set of analyses examined, separately for the two groups, correlations among the three memory systems and between each of the memory systems and performance on the TROG-2. See Table 3. For illustrative purposes, scatterplots showing the relationship between each memory measure and TROG-2 are presented in Figure 2.

**TABLE 3:** **Correlations (Pearson’s r) among memory abilities and receptive grammar for the SLI and TD groups**.

**Variable**	**Group**	**1. Working memory**	**2. Declarative memory**	**3. Procedural memory**
2. Declarative memory	SLI TD	0.333* 0.047	–	–
3. Procedural memory	SLI TD	0.135 –0.039	0.134 0.202	–
4. Receptive grammar	SLI TD	0.372* 0.089	0.469** 0.251	0.036 0.404*

SLI, children with Specific Language Impairment; TD, typically developing children; *p < 0.05; **p < 0.001.

For the TD group, there were no significant associations between the three different memory systems. In terms of associations with receptive grammar, TROG-2 scores correlated significantly with procedural memory only. For the SLI group, there were significant associations between working memory and declarative memory. Moreover, an unlike for the TD group, the TROG-2 correlated with working memory and declarative memory, but not with procedural memory.

Multiple regression analysis was then used to test whether procedural, declarative, and working memory predicted TROG-2 performance, and to examine whether any such predictions differed between the SLI and TD groups. The predictor variables were procedural memory, declarative memory, and working memory as well as a group membership dummy variable whereby 1 = SLI and 0 = TD. Interaction terms created by multiplying the group membership variable with each memory variable were also entered into the regression. For example, to create the “Working Memory × Group” interaction term, scores from the working memory variable were multiplied by group membership. Using this approach, a significant interaction term indicates whether the contribution of the specific memory variable differs between the SLI and TD groups. A summary of the model coefficients is presented in Table 4.

**TABLE 4:** **Regression analysis modeling memory measures as predictors of receptive grammar in the SLI and TD groups**.

**Variable**	**b**	**SE**	**Beta**	***p***
Constant	2.811	0.126		
Working memory	0.015	0.021	0.099	0.482
Declarative memory	0.029	0.023	0.168	0.217
Procedural memory	0.894	0.33	0.287	0.008*
				
Group	–0.297	0.174	–0.202	0.091
Group × working memory	0.026	0.032	0.108	0.410
Group × declarative memory	0.041	0.035	0.15	0.239
Group × procedural memory	–1.037	0.518	–0.202	0.048*

*p < 0.05.

The model was found to be a significant predictor of TROG-2 scores, accounting for 51.6% of variance in the outcome variability [*F* (7, 83) = 12.658, *p* < 0.001, *R*^2^ = 0.516]. Inspection of coefficient values presented in Table 4 shows that the procedural memory predictor variable was statistically significant. The Group × procedural memory Interaction was also found to be statistically significant. Given that children with SLI may have phonological deficits (though this is less likely in the age group of this study, i.e., about 9 years old, than in research involving younger children, Molfese et al., 2010) we repeated the regression analysis while also controlling for phonological abilities, by additionally including in the model performance on the non-word recall task of the WMTB-C. The findings were virtually unchanged. The model explained a similar amount of variance (*R*^2^ = 0.521), the procedural memory predictor variable continued to be statistically significant as was the Group × Procedural Memory interaction, with no other significant effects.

To investigate the interaction further, the above regression analysis was undertaken separately for each group (while also removing the interaction terms with group). For the TD group the model was significant, accounting for 20.3% of the variance [*F* (3, 42) = 3.558, *p* = 0.022, *R*^2^ = 0.203]. In this model the only significant predictor was procedural memory (β = 0.374, *p* = 0.011; declarative memory β = 0.172, *p* = 0.230; working memory β = 0.096, *p* = 0.492). The model was also significant for the SLI group [*F* (3, 41) = 5.180, *p* = 0.004, *R*^2^ = 0.275], explaining 27.5% of the variance. Unlike the TD group, procedural memory was not a significant predictor (β = –0.050, *p* = 0.714). In contrast, for children with SLI, declarative memory was significant (β = –0.393, *p* = 0.008). Working memory also made a contribution, but this variable fell short of statistical significance (β = 0.248, *p* = 0.088). As above for the full model, we repeated the regression analyses while also controlling for phonological abilities, by additionally including in the model performance on the non-word recall task of the WMTB-C. The findings were virtually unchanged. Procedural memory remained the only statistically significant predictor of receptive grammar for TD children, while declarative memory remained the only statistically significant memory system predictive of receptive grammar in children with SLI.

## Discussion

This study examined the relationship between receptive grammar and procedural, declarative, and working memory in children with SLI and TD children. Children with SLI performed worse than the TD children on all the three memory measures as well as receptive grammar. The significant differences between the SLI and TD groups observed on receptive grammar replicate findings of previous studies (e.g., Rice et al., 1998; Conti-Ramsden et al., 2001; Montgomery and Evans, 2009). Indeed, grammatical deficits are the hallmark phenotype of SLI. We also replicated evidence that, when examined independently, verbal working memory, verbal declarative memory and non-verbal procedural memory are impaired in children with SLI (Archibald and Gathercole, 2006a; Lum et al., 2012). It needs to be noted, however, that measures of declarative memory do not appear to differ between the SLI and TD groups once factors such as working memory are controlled for (Lum et al., 2012, 2015).

Importantly, we found striking differences between children with SLI and their TD peers regarding how the three memory measures predicted receptive grammar, in multiple regression models. Procedural memory was the only significant predictor of receptive grammar in TD children. In contrast, for children with SLI, the only significant predictor was declarative memory. This pattern of findings—that is, only procedural memory predicting grammar in TD and only declarative memory in SLI—remained after controlling for phonological abilities. These results indicate that different memory systems are associated with receptive grammar in children with SLI and TD children.

These findings are consistent with the predictions of the DP model and PDH that TD children depend largely on procedural memory for grammar, whereas children with SLI should tend to rely on declarative memory, which is hypothesized to play a compensatory role in the face of procedural and grammatical deficits (Ullman, 2004, in press; Ullman and Pierpont, 2005; Ullman and Pullman, 2015). Thus, the declarative memory system appears to at least partially take over grammatical processing in children with SLI. Moreover, this appears to take place by age 9–10, and perhaps earlier. Indeed, given their dependence on declarative memory at this age, it seems likely that aspects of grammar were learned in this system at an earlier age.

Thus, in the TROG-2 task, in which children with SLI hear a sentence and try to understand it in order to choose the correct picture, they are likely to be relying on declarative memory-based mechanisms, such as chunking of linguistic forms, learning explicit rules, or relying on the semantics of the sentence (Ullman, 2001b, 2004, 2005, 2006, in press; Ullman and Pullman, 2015). Unlike their TD peers, children with SLI do not seem to be able to rely or benefit from the automatic, implicit identification of grammatical patterns using procedural memory, because this system in these children is at least somewhat dysfunctional. We suggest that processing grammatical aspects of language via compensatory declarative memory, although possible to some extent and likely to begin relatively early in development, is generally more burdensome and less efficient for children with SLI (Ullman and Pullman, 2015), affecting how well they can perform on tasks such as the TROG-2. Thus, despite such compensation, their grammar still tends to remain impaired. For further discussion, see Ullman and Pullman (2015). Future research, in particular employing longitudinal paradigms, is needed to examine such memory processes developmentally in children with SLI.

In addition, verbal working memory appears to be somewhat predictive of receptive grammar in children with SLI, but not in their TD peers, at least at this stage of development (Hesketh and Conti-Ramsden, 2013). Consistent with the DP model and the PDH, children with SLI may have not only procedural memory deficits but also (though perhaps less consistently; Ullman and Pierpont, 2005) working memory deficits, as both capacities rely on frontal/basal ganglia circuits. Working memory could affect receptive grammar in SLI in more than one way. First of all, evidence suggests that working memory may be a gateway to declarative memory, whereby the former maintains at least some information both before it enters the latter, as well as after such information is recalled (e.g., before or during further processing; Lum et al., 2015; Ullman, in press). Thus, deficits of working memory may be expected to impede both the learning of grammatical and other information in declarative memory, as well as the recall and use of that information later on (Ullman and Pullman, 2015). For example, information in deficient working memory might decay before it has a chance to be learned in declarative memory or after it has been retrieved from this system but before it undergoes processing. Indeed, children with SLI only appear to show declarative memory (learning) impairments if they have verbal working memory deficits (Lum et al., 2015). Thus, working memory deficits should slow down the learning of grammatical (and non-grammatical) knowledge or strategies in declarative memory, as well as impede the use of that information during processing (Ullman and Pullman, 2015). TD children should be less susceptible to such effects not only because they do not have working memory deficits, but also because their grammar does not depend (or depends less) on declarative memory. Given the available evidence, however, it is difficult to know what is causing what in relation to working and declarative memory. It is likely that the effects are bidirectional. Research has documented effects in the other direction, i.e., the ability to retrieve information from declarative memory also appears to be an important component of performance on working memory tasks (Shipstead et al., 2014; Unsworth et al., 2014). Further research addressing these issues is certainly warranted.

Note that a particularly important role for declarative (and perhaps working) memory in grammar in SLI does not preclude an additional role for procedural memory. After all, the compensatory role for declarative memory should depend on the extent to which procedural memory is impaired. Indeed, some evidence suggests that procedural memory deficits are associated with grammatical deficits in SLI (Hedenius et al., 2011). Interestingly, in that study only a measure reflecting the consolidation of procedural learning over the course of about 3 days predicted grammatical abilities. This may explain why the measure of procedural learning in the present study (which measured sequence knowledge only immediately after learning) did not show associations with grammar. Future studies should examine this issue. It is also important to acknowledge that in this study the performance of children with SLI and TD children were more similar on procedural memory than working and declarative memory. Why then should children with SLI “give up” using procedural memory in grammatical processing? Part of the explanation is also a limitation of this investigation. Recall that we examined only a non-verbal measure of procedural memory. Although we argue that this is sufficient for examining the relationship between procedural memory and grammar, it may be the case that procedural memory is not uniformly impaired across non-verbal versus verbal domains. Indeed, it may be the case that in SLI it is *verbal* procedural memory that is particularly dysfunctional and it is verbal procedural memory impairments that explain why children with SLI “abandon” this memory system for grammatical processing (and we argue, as above, this is likely to occur early in development). Future studies would benefit from the examination of verbal procedural memory and its relation to grammar, in comparison to the predictiveness of verbal working and declarative memory tasks.

This investigation has limitations that may be usefully addressed in future research. First, we limited our investigation to behavior, and did not examine the neural bases of the memory deficits and potential compensatory memory mechanisms in SLI. This could be addressed in research that includes behavioral as well as neuro-imaging or electrophysiological techniques (also see Ullman and Pullman, 2015). Second, we did not examine which particular declarative memory compensatory strategies the children with SLI were likely to be using when confronted with processing sentences in the TROG-2. Although some previous research suggests that both chunking and explicit rules play a role (for a review, see Ullman and Pullman, 2015), future research can further elucidate these mechanisms. Third, non-word recall was the only measure of phonological processing used in this study. Future research would benefit from the inclusion of other measures of phonological abilities in the study of memory and language in SLI. Finally, this investigation was cross-sectional. It examined associations and posited a more fundamental or underlying role to memory processes in language learning in children with and without SLI. The design of the study did not allow the examination of causal relationships which could be usefully addressed in future longitudinal research. For example, might procedural memory deficits or declarative memory strengths at a very early age in children with SLI predict the level of grammatical deficits as the child develops?

## Concluding Remarks

This study shows that receptive grammar is associated with different memory systems in children with SLI and TD children. It is associated with only procedural memory in TD children, and mainly with declarative memory in children with SLI, perhaps with some influence of working memory. Consistent with the DP model and PDH, these associations suggest that different memory systems are involved in receptive grammar in children with SLI and their TD peers of about age 9–10 years. Whereas TD children seem to depend on procedural memory for receptive grammar, children with SLI appear to rely on declarative memory. These findings strengthen the view that children with SLI partially compensate for their grammatical deficits by relying on declarative memory (Ullman and Pierpont, 2005; Ullman and Pullman, 2015).

These findings have potential practical implications (for more discussion, see Ullman and Pullman, 2015). For example, in children with SLI improvements in grammar should be observed following interventions which harness cognitive and behavioral strategies that depend on declarative memory, such as “chunking” of complex forms, or techniques that improve learning in this system, such as “spaced” as opposed to “massed” presentation (Ullman and Pullman, 2015). Finally, of both practical and research interest is the fact that sleep appears to promote consolidation in declarative memory (Marshall and Born, 2007; Ullman and Pullman, 2015). The findings of the present investigation suggest that research examining sleep and language learning may reveal that the amount of sleep may be an important variable for the development of compensatory declarative memory strategies in children with SLI.

## Author Contributions

GCR conceived of the study, developed the design, directed the data collection, contributed to the data analysis, the theoretical framework and interpretation of results and the drafting of the manuscript. JL contributed to the conception of the study, the development of the design, the data collection, the data analysis and the theoretical framework and interpretation of the results and the drafting of the manuscript. MU conceived the theory underpinning this study, developed the theoretical framework for the study, contributed to the data analysis, the interpretation of results and the drafting of the manuscript. All authors read and approved the final manuscript.

### Conflict of Interest Statement

The authors declare that the research was conducted in the absence of any commercial or financial relationships that could be construed as a potential conflict of interest.

## References

[B1] Ackerman P. L. Cianciolo A. T. (2000). Cognitive, perceptual-speed, and psychomotor determinants of individual differences during skill acquisition. J. Exp. Psychol. Appl. 6, 259–290. 10.1037/1076-898X.6.4.259 11218338

[B2] Adams A. Gathercole S. E. (1996). Phonological working memory and spoken language development in young children. Q. J. Exp. Psychol. 49A, 216–233. 10.1080/713755610

[B3] Alloway T. P. Archibald L. (2008). Working memory and learning in children with developmental coordination disorder and specific language impairment. J. Learn. Disabil. 41, 251–262. 10.1177/0022219408315815 18434291

[B4] American Psychiatric Association. (2000). Diagnostic and Statistical Manual of Mental Disorders: DSM-IV-TR, 4th Edn. Washington, DC: American Psychiatric Association.

[B5] Archibald L. Gathercole S. (2006a). Short-term and working memory in specific language impairment. Int. J. Lang. Commun. Disord. 41, 675–693. 10.1080/13682820500442602 17079222

[B6] Archibald L. M. Gathercole S. E. (2006b). Visuospatial immediate memory in specific language impairment. J. Speech Lang. Hear. Res. 49, 265–277. 10.1044/1092-4388(2006/022) 16671843

[B7] Archibald L. Gathercole S. E. (2007). The complexities of complex memory span: storage and processing deficits in specific language impairment. J. Mem. Lang. 57, 177–194. 10.1016/j.jml.2006.11.004

[B8] Baddeley A. (2003). Working memory: looking back and looking forward. Nat. Rev. Neurosci. 4, 829–839. 10.1038/nrn1201 14523382

[B9] Baddeley A. A. Hitch G. (1974). “Working memory,” in Psychology of Learning and Motivation, Vol. 8, ed. Bower G. A. (New York: Academic Press), 47–89.

[B10] Bishop D. (2003). Test for Reception of Grammar-2, 2nd Edn. London: Pearson Assessment.

[B11] Bishop D. V. M. (2014). Ten questions about terminology for children with unexplained language problems. Int. J. Lang. Commun. Disord. 49, 381–415. 10.1111/1460-6984.12101 25142090 PMC4314704

[B12] Bishop D. Adams C. Norbury C. (2006). Distinct genetic influences on grammar and phonological short-term memory deficits: evidence from 6-year-old twins. Genes Brain Behav. 2, 158–169. 10.1111/j.1601-183X.2005.00148.x 16507007

[B13] Botting N. Conti-Ramsden G. (2001). Non-word repetition and language development in children with specific language impairment (SLI). Int. J. Lang. Commun. Disord. 36, 421–432. 10.1080/13682820110074971 11802495

[B14] Cohen J. (1997). Children’s Memory Scales. London: The Psychological Corporation.

[B15] Conti-Ramsden G. (2014). What should we call children who struggle to talk? Taking a developmental, global perspective of diagnostic labels-reflections on Bishop (2014). Commentary on Bishop, D. V. M., Ten questions on terminology for children with unexplained language problems. Int. J. Lang. Commun. Disord. 49, 381–415. 25142090 10.1111/1460-6984.12101 PMC4314704

[B16] Conti-Ramsden G. Botting N. Faragher B. (2001). Psycholinguistic markers for specific language impairment (SLI). J. Child Psychol. Psychiatry 42, 741–748. 10.1111/1469-7610.00770 11583246

[B17] Conti-Ramsden G. St Clair M. Pickles A. Durkin K. (2012). Developmental trajectories of verbal and nonverbal skills in individuals with a history of SLI: from childhood to adolescence. J. Speech Lang. Hear. Res. 55, 1716–1735. 10.1044/1092-4388(2012/10-0182) 22562827

[B18] Cowan N. (2012). Working Memory Capacity. Hove: Psychology Press.

[B19] Ellis Weismer S. Evans J. Hesketh L. J. (1999). An examination of verbal working memory capacity in children with specific language impairment. J. Speech Lang. Hear. Res. 42, 1249–1260. 10.1044/jslhr.4205.1249 10515519

[B20] Evans J. L. Saffran J. R. Robe-Torres K. (2009). Statistical learning in children with specific language impairment. J. Speech Lang. Hear. Res. 52, 321–335. 10.1044/1092-4388(2009/07-0189) 19339700 PMC3864761

[B21] Gathercole S. E. Baddeley A. D. (2014). Working Memory and Language Processing. Hove: Psychology Press.

[B22] Hedenius M. Persson J. Tremblay A. Adi-Japha E. Veríssimo J. Dye C. D. (2011). Grammar predicts procedural learning and consolidation deficits in children with specific language impairment. Res. Dev. Disabil. 32, 2362–2375. 10.1016/j.ridd.2011.07.026 21840165 PMC3191257

[B23] Hesketh A. Conti-Ramsden G. (2013). Memory and language in middle childhood for individuals with a history of specific language impairment. PLoS ONE 8:e56314. 10.1371/journal.pone.0056314 23409172 PMC3567067

[B24] Hill P. Hogben J. Bishop D. (2005). Auditory frequency discrimination in children with specific language impairment: a longitudinal study. J. Speech Lang. Hear. Res. 48, 1136–1146. 10.1044/1092-4388(2005/080) 16411802

[B25] Karuza E. A. Newport E. L. Aslin R. N. Starling S. J. Tivarus M. E. Bavelier D. (2013). The neural correlates of statistical learning in a word segmentation task: an fMRI study. Brain Lang. 127, 46–54. 10.1016/j.bandl.2012.11.007 23312790 PMC3750089

[B26] Kidd E. (2013). The role of verbal working memory in children’s sentence comprehension: a critical review. Topics Lang. Disord. 33, 208–223. 10.1097/TLD.0b013e31829d623e

[B27] Lum J. A. Conti-Ramsden G. Morgan A. T. Ullman M. T. (2014). Procedural learning deficits in specific language impairment (SLI): a meta-analysis of serial reaction time task performance. Cortex 51, 1–10. 10.1016/j.cortex.2013.10.011 24315731 PMC3989038

[B28] Lum J. A. G. Conti-Ramsden G. Page D. Ullman M. T. (2012). Working, declarative and procedural memory in specific language impairment. Cortex 48, 1138–1154. 10.1016/j.cortex.2011.06.001 21774923 PMC3664921

[B29] Lum J. A. Ullman M. T. Conti-Ramsden G. (2015). Verbal declarative memory impairments in specific language impairment are related to working memory deficits. Brain Lang. 142, 76–85. 10.1016/j.bandl.2015.01.008 25660053 PMC4346274

[B30] Marshall L. Born J. (2007). The contribution of sleep to hippocampus-dependent memory consolidation. Trends Cogn. Sci. 11, 442–450. 10.1016/j.tics.2007.09.001 17905642

[B31] Marton K. Schwartz R. G. (2003). Working memory capacity and language processes in children with specific language impairment. J. Speech Lang. Hear. Res. 46, 1138–1153. 10.1044/1092-4388(2003/089) 14575348

[B32] McArthur G. Bishop D. (2004). Frequency discrimination deficits in people with specific language impairment: reliability, validity, and linguistic correlates. J. Speech Lang. Hear. Res. 47, 527–541. 10.1044/1092-4388(2004/041) 15212566

[B33] Molfese D. Maguire M. Molfese V. Pratt N. Ratajczak E. Fentress L. (2010). “Phonological, lexical, syntactic, and semantic disorders in children,” in Concise Encyclopedia of Brain and Language, ed. Whitaker H. A. (Oxford: Elsevier), 535–545.

[B34] Montgomery J. W. (1995). Sentence comprehension in children with specific language impairment: the role of phonological working memory. J. Speech Lang. Hear. Res. 38, 187–199. 10.1044/jshr.3801.187 7731209

[B35] Montgomery J. W. Evans J. O. (2009). Complex sentence comprehension and working memory in children with Specific Language Impairment. J. Speech Lang. Hear. Res. 52, 269–288. 10.1044/1092-4388(2008/07-0116) 18723601 PMC4684953

[B36] Montgomery J. W. Magimairaj B. M. Finney M. C. (2010). Working memory and specific language impairment: an update on the relation and perspectives on assessment and treatment. Am. J. Speech Lang. Pathol. 19, 78–94. 10.1044/1058-0360(2009/09-0028) 19948760

[B37] Montgomery J. W. Polunenko A. Marinelli S. A. (2009). Role of working memory in children’s understanding spoken narrative: a preliminary investigation. Appl. Psycholinguist. 30, 485–509. 10.1017/s0142716409090249

[B38] Nissen M. J. Bullemer P. (1987). Attentional requirements of learning: evidence from performance measures. J. Cogn. Psychol. 19, 1–32. 10.1016/0010-0285(87)90002-8

[B39] Norbury C. F. Bishop D. V. M. Briscoe J. (2001). Production of English finite verb morphology: a comparison of SLI and mild-moderate hearing impairment. J. Speech Lang. Hear. Res. 44, 165–178. 10.1044/1092-4388(2001/015) 11218100

[B40] Norbury C. F. Bishop D. V. Briscoe J. (2002). Does impaired grammatical comprehension provide evidence for an innate grammar module? Appl Psycholinguist. 23, 247–268. 10.1017/S0142716402002059

[B41] Pickering S. J. Gathercole S. E. (2001). Working Memory Test Battery for Children (WMTB-C). London: Pearson Assessment.

[B42] Reilly S. Bishop D. V. M. Tomblin B. (2014a). Terminological debate over language impairment in children: forward movement and sticking points. Int. J. Lang. Commun. Disord. 49, 452–462. 10.1111/1460-6984.12111 25142092 PMC4312775

[B43] Reilly S. Tomblin B. Law J. McKean C. Mensah F. K. Morgan A. (2014b). Specific language impairment: a convenient label for whom? Int. J. Lang. Commun. Dis. 49, 416–451. 10.1111/1460-6984.12102 25142091 PMC4303922

[B44] Rice M. Wexler K. Hershberger S. (1998). Tense over time: the longitudinal course of tense acquisition in children with specific language impairment. J. Speech Lang. Hear. Res. 41, 1412–1431. 10.1044/jslhr.4106.1412 9859895

[B45] Semel E. Wiig E. H. Secord W. A. (2003). Clinical Evaluation of Language Fundamentals UK Standardisation (CELF-4 UK), 4th Edn. San Antonio: The Psychological Corporation.

[B46] Shipstead Z. Lindsey D. R. Marshall R. L. Engle R. W. (2014). The mechanisms of working memory capacity: primary memory, secondary memory, and attention control. J. Mem. Lang. 72, 116–141. 10.1016/j.jml.2014.01.004

[B47] Thomas K. M. Hunt R. H. Vizueta N. Sommer T. Durston S. Yang Y. (2004). Evidence of developmental differences in implicit sequence learning: an fMRI study of children and adults. J. Cogn. Neurosci. 16, 1339–1351. 10.1162/0898929042304688 15509382

[B48] Tomblin J. B. Records N. L. Buckwalter P. Zhang X. Smith E. O’Brien M. (1997). Prevalence of specific language impairment in kindergarten children. J. Speech Lang. Hear. Res. 40, 1245–1260. 10.1044/jslhr.4006.1245 9430746 PMC5075245

[B49] Ullman M. T. (2001a). A neurocognitive perspective on language: the declarative/procedural model. Nat. Rev. Neurosci. 2, 717–726. 10.1038/35094573 11584309

[B50] Ullman M. T. (2001b). The declarative/procedural model of lexicon and grammar. J. Psycholinguist. Res. 30, 37–69. 10.1023/A:1005204207369 11291183

[B51] Ullman M. T. (2004). Contributions of memory circuits to language: the declarative/procedural model. Cognition 92, 231–270. 10.1016/j.cognition.2003.10.008 15037131

[B52] Ullman M. T. (2005). “A cognitive neuroscience perspective on second language acquisition: the declarative/procedural model,” in Mind and Context in Adult Second Language Acquisition: Methods, Theory and Practice, ed. Sanz C. (Washington, DC: Georgetown University Press), 141–178.

[B53] Ullman M. T. (2006). The declarative/procedural model and the shallow-structure hypothesis. Appl Psycholinguist. 27, 97–105. 10.1017/S014271640606019X

[B54] Ullman M. T. (in press). “The declarative/procedural model: a neurobiological model of language learning, knowledge and use,” in The Neurobiology of Language, eds Hickok G. Small S. A. (Elsevier) Available at: http://store.elsevier.com/Neurobiology-of-Language/isbn-9780124077942/

[B55] Ullman M. T. Pierpont E. I. (2005). Specific language impairment is not specific to language: the procedural deficit hypothesis. Cortex 41, 399–433. 10.1016/S0010-9452(08)70276-4 15871604

[B56] Ullman M. T. Pullman M. Y. (2015). A compensatory role of declarative memory in neurodevelopmental disorders. Neurosci. Biobehav. Rev. 51, 205–222. 10.1016/j.neubiorev.2015.01.008 25597655 PMC4359651

[B57] Unsworth N. Fukuda K. Awh E. Vogel E. K. (2014). Working memory and fluid intelligence: capacity, attention control, and secondary memory retrieval. Cogn. Psychol. 71, 1–26. 10.1016/j.cogpsych.2014.01.003 24531497 PMC4484859

[B58] Wechsler D. (1999). Wechsler Abbreviated Scale of Intelligence (WASI). San Antonio: The Psychological Corporation.

[B59] Zhuang P. Dang N. Warzeri A. Gerloff C. Cohen L. Hallett M. (1998). Implicit and explicit learning in an auditory serial reaction time task. Acta Neurol. Scand. 97, 131–137. 10.1111/j.1600-0404.1998.tb00622.x 9517864

